# Information theory reveals physiological manifestations of COVID-19 that correlate with symptom density of illness

**DOI:** 10.3389/fnetp.2024.1211413

**Published:** 2024-06-14

**Authors:** Jacob M. Ryan, Shreenithi Navaneethan, Natalie Damaso, Stephan Dilchert, Wendy Hartogensis, Joseph L. Natale, Frederick M. Hecht, Ashley E. Mason, Benjamin L. Smarr

**Affiliations:** ^1^ Halıcıoğlu Data Science Institute, University of California, San Diego, La Jolla, CA, United States; ^2^ Department of Bioengineering, University of California, San Diego, La Jolla, CA, United States; ^3^ MIT Lincoln Laboratory, Massachusetts Institute of Technology, Lexington, MA, United States; ^4^ Department of Management, Zicklin School of Business, Baruch College, The City University of New York, New York, NY, United States; ^5^ Osher Center for Integrative Health, University of California, San Francisco, San Francisco, CA, United States

**Keywords:** information theory, COVID-19, wearable devices, network physiology, data science

## Abstract

Algorithms for the detection of COVID-19 illness from wearable sensor devices tend to implicitly treat the disease as causing a stereotyped (and therefore recognizable) deviation from healthy physiology. In contrast, a substantial diversity of bodily responses to SARS-CoV-2 infection have been reported in the clinical milieu. This raises the question of how to characterize the diversity of illness manifestations, and whether such characterization could reveal meaningful relationships across different illness manifestations. Here, we present a framework motivated by information theory to generate quantified maps of illness presentation, which we term “manifestations,” as resolved by continuous physiological data from a wearable device (Oura Ring). We test this framework on five physiological data streams (heart rate, heart rate variability, respiratory rate, metabolic activity, and sleep temperature) assessed at the time of reported illness onset in a previously reported COVID-19-positive cohort (N = 73). We find that the number of distinct manifestations are few in this cohort, compared to the space of all possible manifestations. In addition, manifestation frequency correlates with the rough number of symptoms reported by a given individual, over a several-day period prior to their imputed onset of illness. These findings suggest that information-theoretic approaches can be used to sort COVID-19 illness manifestations into types with real-world value. This proof of concept supports the use of information-theoretic approaches to map illness manifestations from continuous physiological data. Such approaches could likely inform algorithm design and real-time treatment decisions if developed on large, diverse samples.

## 1 Introduction

The COVID-19 pandemic spurred many efforts to develop algorithms that take in wearable sensor data (as in Oura Ring: Mason et al., 2022; FitBits; Liu et al., 2022; Mishra et al., 2020; Nestor et al., 2021; Shapiro et al., 2020; Natarajan et al., 2020; Apple Watches; Hirten et al., 2021; Cleary et al., 2021; and Whoop; Miller et al., 2020) and give back alerts for possible infections. These algorithms are based on the idea that physiology changes during illness, and that these changes, captured by wearable devices, can be used to train machine learning algorithms. However, while some physiological changes are anticipated with most illnesses—such as elevated temperature and heart rate (Li et al., 2017) —it is obvious that not all COVID-19 patients manifest illness in the same way (Alimohamadi et al., 2020; Klein et al., 2022). Methods are therefore needed to quantify the extent to which different “manifestations” can be identified. This would allow algorithms to be trained more precisely by separating manifestations into different training pools. It might also impact care decisions if it is found that different manifestations at the onset of illness respond to different treatments, or are associated with different outcomes.

Relatedly, another common assumption is that each physiological system measured is providing an independent measurement about only itself. Instead physiological systems are known to influence each other, acting as a network where organs or system components (nodes) influence the activity of other nodes by way of internal signaling (edges) (e.g., Grant et al., 2018; Sorelli et al., 2022; Zhang et al., 2022; Campanaro et al., 2023; Hasselman et al., 2023). Without this conceptual framework of a physiological network, health algorithms commonly assess the weight of change in each physiological system as an independent measure of change, and classify illness as the presence of substantial change above some overall threshold, but where the specific combinations of change-by-system might not be counted as informative. That is, one person might be classified by an algorithm as suffering illness due to changes in heart rate despite having a low temperature, where someone else might be classified as suffering illness due to elevated temperature despite less change in their heart rate than the first person. If all we know is that both people appear ill, then we lose the information about the different physiological manifestations that took them past that threshold of detection. However, with the physiological network framework, it might be possible to use multimodal sensors to detect different conformations or states from the resulting network, providing information beyond merely the sum amplitude of change from each individual sensor modality (physiological system). Since different treatments will interact with different physiological systems, and since different patterns of infection might be detected through comparison of those manifestations, we sought here to test whether those different manifestations are indeed detectable and if so, non-random, using data gathered during the COVID-19 pandemic of 2020.

We hypothesize that there is more than one identifiable way in which COVID-19 illness manifests across individuals, and that appropriate tests could add numerical weight to determining if these differences are random, or cohere into subtypes of manifestation in physiological data, which might then be correlated to aspects of the illness (in our case, symptom density of the participant with COVID-19). With the hypothesis that there is more than one identifiable way in which COVID-19 illness manifests across individuals, we aim here to present a generalizable framework to test whether there are certain manifestations that occur more frequently than others, and whether the physiological manifestations detected correlate with other aspects of the illness experience. We are not aware of a commonly accepted method of quantifying these physiological changes that occur during illness, so the point of this study was to developed a simple method as a proof-of-concept to do so, relying on data from a previously published cohort of confirmed COVID-19 cases gathered across 2020 (Mason et al., 2022). To do so, we draw on two information-theoretic paradigms—binning or categorizing many-valued variables into coarse stratifications of only a few “summary” elements, and observing how evenly the values of one such stratified variable “spread” in trying to predict another.

## 2 Methods

### 2.1 Subjects and data collection

The N = 73 participants included in this case study are a group of individuals from a larger data set from the TemPredict study, as described in detail in Mason et al. (2022), in which participants provided data collected with the commercially available wearable device Oura Ring (Oura Health Oy, Oulu, Finland). Additional details on specific data as well as the recruitment and exclusion criteria of the initial cohort are outlined in Mason et al. (2022); however, here we additionally outline relevant details of the subset used in this study. The original TemPredict study contained 63,153 individuals, 704 of whom self-reported COVID-19 in 2020. Of these 73 had confirmatory tests and also consistent data collection throughout several weeks surrounding their illness. Within the 73, 28 were male and 44 were female, 1 declined to report gender. For the purposes of this manuscript, the data contain five physiological data streams: heart rate (HR), heart rate variability (HRV) defined by the root mean squared of successive differences in heart beats (RMSSD), respiratory rate (RR), metabolic activity (MET), and distal skin temperature (T), which are provided from the Oura App. These were summarized into 30 min means, with a 15 min step size, resulting in summary values per 15 min for each physiological data stream. MET, provided in units of metabolic equivalents (rest being 1 and higher values being multiples of this value, whereas in much of actigraphy, rest is 0 and absolute activity, as in steps per minute, is used instead; the exact formula is proprietary and not known to the authors) was used across the whole 24 h, while HR, HRV, and RR were only available to us during sleep times. T was similarly divided into wake and sleep times, and we reference only sleep T here. Participants were also given the option to respond to daily surveys which included the opportunity to report COVID-19 symptoms along with means of testing and case confirmation. The present set of 73 participants represent the subset of participants from the larger study who provided both confirmed COVID-19 diagnoses and maximal data quality. The quality of individuals’ data was judged by the completeness of their wearable-device-derived temperature data, i.e., how often data were missing in the period of approximately 14 days surrounding their COVID-19 diagnoses. Moreover, these participants had no anomalies in their physiological data streams.

### 2.2 Baseline period

**FIGURE 1:**
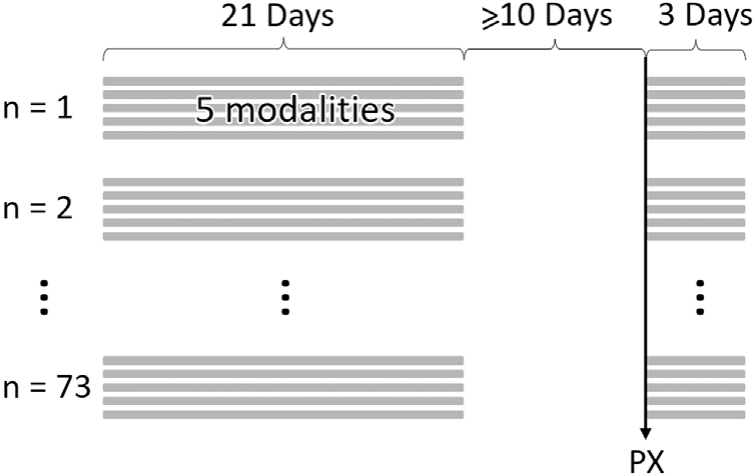
Example data structure and timeline for N = 73 samples. At least 10 days between physiological onset of symptoms (PX) and baseline samples. Baseline samples can be non-consecutive 21 days. Illness period begins on PX and ends 3 days later. Each day of data produces 5 physiological data streams (modalities).

For each participant, an individually-curated period equivalent to 21 total days of high-resolution data (sampling rate 15 min) at least 10 days before their COVID-19 diagnosis was created to represent their physiology prior to illness. This period was not necessarily a continuous 3 weeks for everyone due to gaps in data availability. Hence, we call this a curated three-week baseline period. In the cases where the 21 days’ worth of data were from a non-continuous three-week period, the curation ensured a proportionate representation of both weekend and weekday records (Figure 1).

### 2.3 Illness window

In order to define an “illness window” for each individual from which to draw samples for comparison with baseline physiology, we made use of the physiological disruption (PX) dates used during the first TemPredict study (Mason et al., 2022). Using two of the five physiological data streams (HR and RR), the PX computation attempts to impute the maximally discernible deviation from baseline physiology in the periods leading up to, and including, an individual’s diagnosis date, as detailed in (Mason et al., 2022).

We defined the illness window for each individual as a consecutive, three-day period starting on the date of physiological disruption onset (PX date, defined further in Mason et al., 2022) and ending 2 days following PX (i.e., PX+2) (Figure 1). Three days were chosen so as to provide enough measurement points to observe any potential physiological disruptions, without including so many points that the detection of a distribution shift from baseline would become diluted due to large-scale averaging.

### 2.4 Manifestation labels

Changes to each physiological data stream for each individual were assigned a change label of +1, 0, or −1 at the individual’s PX date. To generate these labels, we compared our baseline periods to our illness windows and performed a series of Mann-Whitney U Tests for each individual, independently for each physiological data stream, provided that stream contained at least 15 observations in the baseline period and also 15 in the illness window periods. There was no minimum spacing applied to these 15 observations. If the distribution of values for a given physiological data stream during the illness window demonstrated an overall shift down from the values in the baseline period through stochastic dominance (McFadden, 1989) in the Mann-Whitney *U* Test, we assigned that stream a label of −1 for the participant in question. Similarly, if the distribution of the physiological data stream values in the illness window demonstrated an overall shift upward from the values in the baseline, we assigned that stream a label of +1 for that participant. Finally, if the distribution of values in the illness window represented no statistically significant shift from the values in baseline, we assigned that stream a label of 0 for that participant. For those cases in which a participant had less than 15 observations for a given physiological data stream, in either the baseline or illness window period, we assigned that stream a label of NaN (i.e., “Not a Number”) for that participant.

The above process of label generation was applied to all physiological data streams for each of the 73 participants, creating a five-dimensional vector of labels. We refer to each individual’s resultant vector as a manifestation, because it reflects the full available physiological disruption pattern that was observed at the onset of illness. For labeling and notation convenience, the labels for all participants were then concatenated together as a record of all the manifestations observed in this particular cohort (*i.e*., a 73 × 5 matrix).

### 2.5 Common/rare manifestations

We categorize a given manifestations as *common* if it appears more than once in our data set, and *rare* if it appears exactly once. The threshold of “one or more-than-one” is a simple choice that avoids the need for any more complex argument in the absence of strong, pre-analytic justification. While a manifestation that shows up only once in this small data set might well appear more frequently in a larger one, this is unverifiable here. A manifestation that shows up more than once in the context of a small sample size is more likely to also be more frequent in a larger data set. Expressed another way, naive estimators of entropy (Bein, 2006) (i.e., those based on observed frequencies of appearance of distinct manifestations) tend to *under*estimate entropies and *over*estimate information conveyed by manifestation frequencies about outcome variables; our categorization-labeling threshold here represents a conscious choice to “err on the safe side” by not assuming that singularly-appearing manifestations will appear with proportionally more-frequent rates in larger datasets.

### 2.6 Most common manifestation: *M*_0_

We define *M*_0_ as the most common manifestation. *M*_0_ appeared in our data set 11 times, and includes an increase in HR, decrease in HRV, increase in RR, increase in MET, and increase in sleep T. It is represented by the vector *M*_0_ = [1, -1, 1, 1, 1], corresponding to each of the aforementioned physiological changes.

### 2.7 Distance

Simply labeling different manifestations, as described above, does not render salient any low-dimensional patterns to which they might collapse—for instance, their spanning of a formal subspace in their shared, five-dimensional vector space. Thus, we developed a custom “distance” metric to quantify the relative separations between manifestations in that original, shared space. This metric was formed by modifying the traditional Hamming distance (Ruan et al., 2017) to accommodate ternary, instead of binary, difference classes.

A weight was assigned to each individual physiological manifestation label according to its element-wise (Hamming-style) separation from the single, most commonly-occurring label *M*_0_. These individual weights were then added together to generate a pairwise, weighted distance for each manifestation from *M*_0_. In particular, a weight of zero (0) was assigned if the change in a given stream was *identical* to the corresponding label for the same stream in *M*_0_. A weight of +1 was assigned for any case in which the single-stream change differed by *one unit* from that of *M*_0_ in either direction. In principle, this would apply to any “1” (representing a physiological-value upshift) or “-1” where there was a “0” in *M*_0_, and a “0” where there was a “1” or “-1” in *M*_0_; in practice, since the *M*_0_ vector contained no “0” entries (i.e., no physiological data stream in the most common manifestation “stayed the same” as its pre-illness value, according to the Mann-Whitney *U* test) a distance value of “+1” could only occur in the latter ways.

A weight of +2 was assigned for a two-unit difference—which occurred when the change in a physiological data stream had the *opposite sign* of the corresponding label in *M*_0_. This slightly stronger weighting was implemented on the assumption that manifestations are more fundamentally different from one another, all else being equal, if a given physiological data stream changed sign—e.g., a relative shift downwards instead of a shift upwards—as opposed to shifting without changing sign (potentially just a difference in amplitude of the same nature of change).

Finally, a weight of 0.4 was assigned to labels that were NaNs, which means that there was not enough data in that stream to calculate a label for that person (see “Manifestation Labels” above). The NaN weights of 0.4 were used to distinguish manifestations that included NaN labels from manifestations that did not; this distance allows disambiguation across distance sums otherwise derived from integers, while being of low amplitude to reflect the uncertainty in actual distance.

### 2.8 Symptom density classes

As described in (Mason et al., 2022), participants reported daily experience of COVID-19 symptoms independent from and in addition to providing physiology data from their wearable device. Based on the total number of unique symptoms each person reported starting at PX and ending 2 days following (PX+2), they were classified as having undergone either *asymptomatic* (0 symptoms), *mild* (1-3 symptoms), or *severe* (4+ symptoms) symptomaticity for the purposes of our analyses. If symptom reports were *missing* for a given participant during the aforementioned window (lack of reports being different from a report of 0 symptoms), this participant was assigned a symptom class of NaN.

### 2.9 Statistical test of scatter

To test the significance of the manifestation categorization relation to symptom density, we ran two Student-T simulations for each symptom density category (Mishra et al., 2019). In the first simulation, we utilized the naive, zeroth-order, maximum entropy distribution, i.e., the assumption that we know nothing about the population’s probabilities and thus each manifestation category has a probability of one half of being associated with the symptom density classification being tested. In the second simulation, we utilized the first-order maximum entropy distribution, with the assumption that the existing probabilities in the sample of manifestation category sizes are the population probabilities and thus each manifestation category’s probability is equal to the proportion of that category’s presence in the sample of *N* = 73. The difference between the two simulations for each symptom density classification was an estimate of the population probability for the manifestation categories.

Both simulations entailed a random draw, with replacement, from a population of the two manifestation categories (common/rare), for a total number of draws equal to the number of appearances in a given symptom density classification.

### 2.10 Sign switches

We defined sign switch in a manifestation as having an opposite label in a physiological data stream comparative to *M*_0_. For example, if a manifestation had a −1 for heart rate, then since *M*_0_ has a +1 for heart rate, this manifestation was said to have a sign switch. However, if that manifestation had a 0 for heart rate, it would not be defined as a manifestation with a sign switch. The manifestations without sign switches are referred to as being from one branch while the manifestations with sign switches are from an alternate branch, as can be seen for common manifestations in Figure 3. The reason for considering manifestations with sign switches to be from an alternate branch is that, if a physiological data stream changes in the opposite way from how it changes in *M*_0_, it is most likely not a “less severe” form of the same manifestation, but must in fact be of a different physiological response. In contrast, if the change in physiology is simply undetectable, there is no clear indication that this manifestation is *caused* in a way that is different to the manifestation represented by *M*_0_, so the manifestation representing this illness would still be considered as part of the *M*_0_ branch.

### 2.11 Principal component analysis

Briefly, Principal Component Analysis identifies the composite dimensions that best account for the variance within a high dimensional data set. This allows for description of variance in a compressed, lower dimensional space in which the new dimensions cross-cut the originals, as in the hypotenuse of a right triangle containing some information from both orthogonal legs. PCA is commonly performed in biochemical and biomolecular analyses, as well as in behavioral and physiological data sets (e.g., Reich et al., 2008; Adolfo et al., 2021). We performed PCA on 39 unique manifestations (2 of the 41 unique manifestations contained NaN values), using the 5-dimensional vectors of physiological change labels (i.e., −1, 0, +1) as inputs. More specifically, we perform PCA on the table with 39 rows (each a manifestation) with five columns (each column being the row’s physiological change label).

### 2.12 Software analyses/tools

All statistical analyses were done with the Python3 *scikit-learn* package except for the Kruskal–Wallis test, which was done with the *scipy.stats* package, and the Student-T simulations, which were custom written using Python3. Figures were built using Python3 except for Figure 3, which was created using Adobe Photoshop.

## 3 Results

### 3.1 Manifestations span non-random, low-dimensional spaces

**FIGURE 2:**
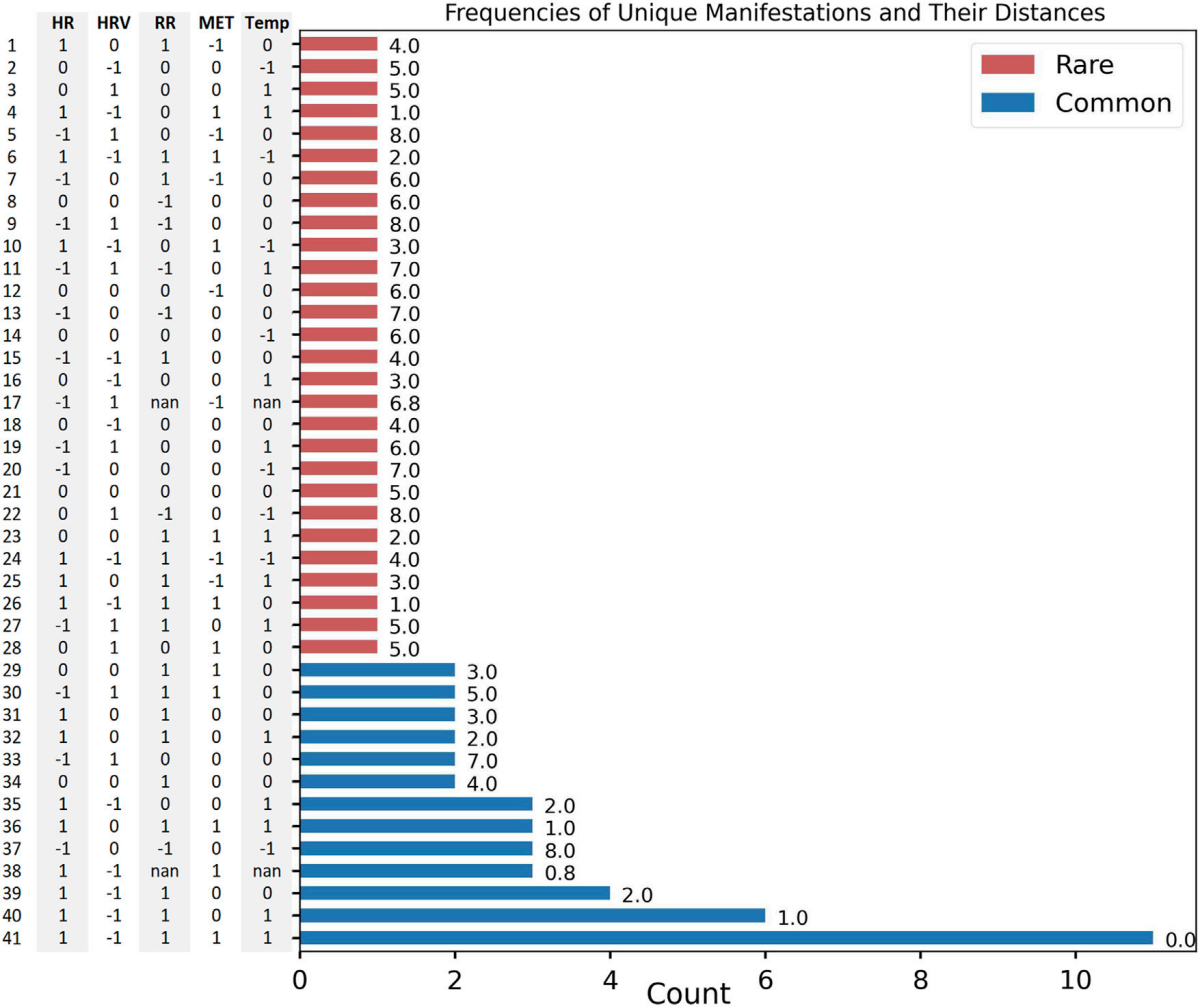
*M* = 41 unique manifestations and their corresponding vectors (*y*-axis). Counts (*x*-axis), Summed Hamming-Inspired distance value (value to the right of the bar). Common (blue), rare (red).

**FIGURE 3:**
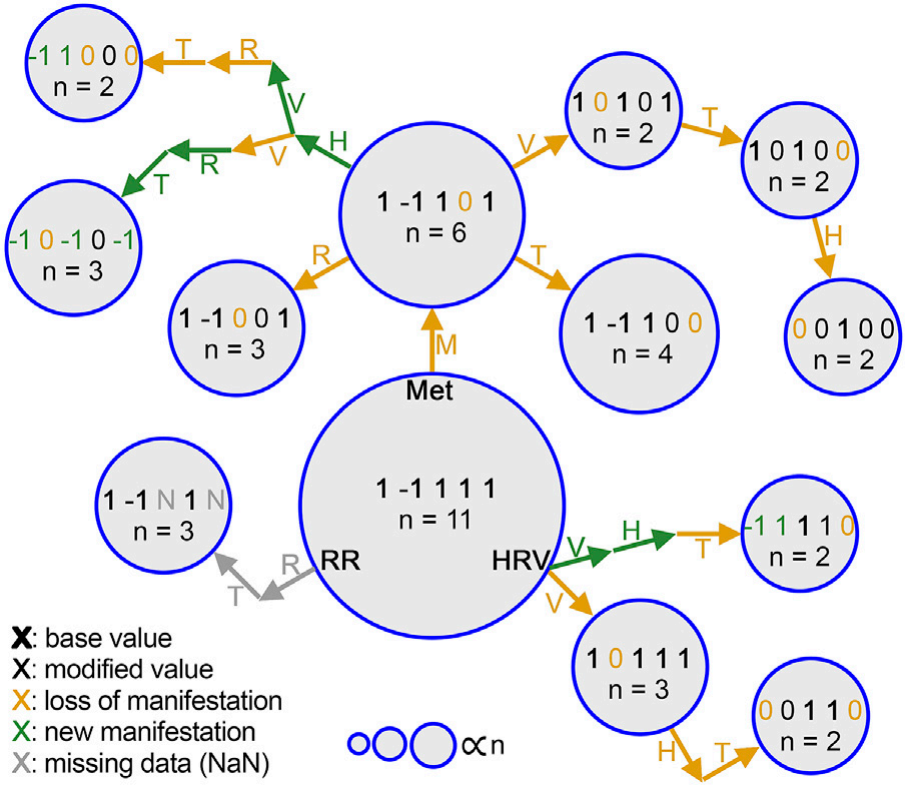
Step-by-step changes in common manifestations from *M*_0_. Each manifestation is represented by a circle, and the area of each circle is proportional to the number of people who had that manifestation (n). Change of each physiological data stream label is denoted by an annotated arrow. heart rate (H), heart rate variability (V), respiratory rate (R), activity (MET, M), temperature (T). Each stream label is colored differently based on whether it indicates a value carried from the previous manifestation (black), reduction to 0 (gold), a sign switch (green), or NaN due to lack of data (gray). Bold indicates a value shared with *M*_0_.

It was previously reported that illness is associated, on average, with a shift in the values of physiological variables compared to baseline (pre-illness) periods (Mason et al., 2022). Here, we quantify differences in each physiological modality, on an individual basis, at the time of reported illness onset (Figure 2) and confirm by inspection that all individuals do *not* manifest their deviations in the same way. From our N = 73 cohort, 41 unique manifestations were observed; the most common of these, to which we attach the label *M*_0_, occurred 11 times (see “Most Common Manifestation” above). As described in Methods, *M*_0_ can be illustrated as [1, −1, 1, 1, 1]. Out of the 41 total manifestations, 13 were categorized as common (observed more than once), and the other 28 were categorized as rare (observed only once). The 13 common manifestations accounted for 45 (61% probability weight) of the 73 participants, whereas the rare manifestations had a collective probability of 39%. Among the common subset, metabolic activity and heart rate variability differed from their respective values in *M*_0_ more often (8 times each) than the other physiological features (Figure 3).

### 3.2 “Common” manifestations cluster around shared physiological characteristics

**FIGURE 4:**
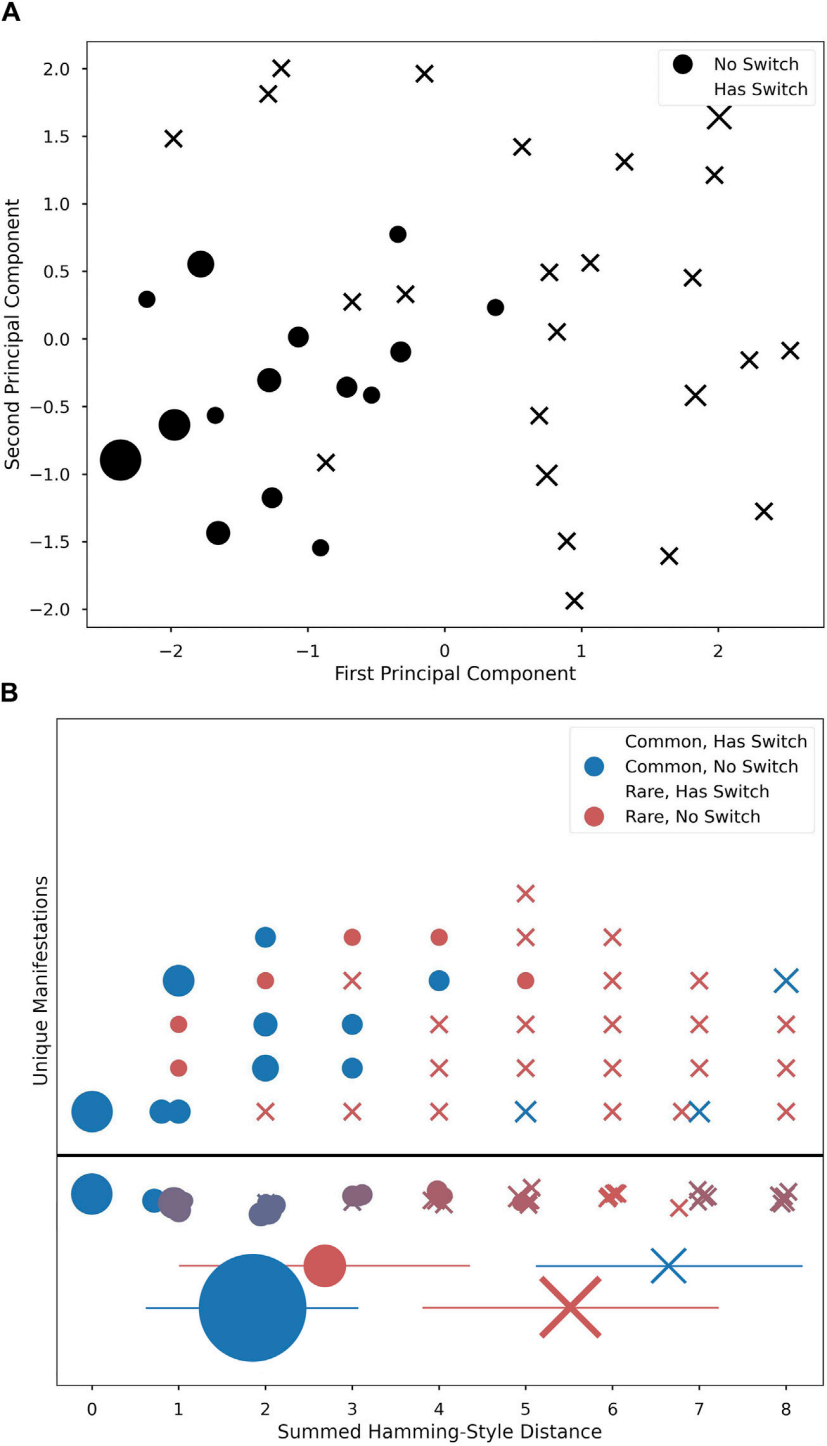
PCA 2-dimensional projection plot of five vector physiological changes **(A)**. Main branch of non-switch manifestations seen in bottom left corner (circles), with more sparse peripheral cases with switches (“X”s). Unique Manifestations **(B)** ordered horizontally by distance and stacked vertically in arbitrary order. Shape marks switch and color marks common/rare. Row below the line is a summation of figures above to represent average ‘color’ (i.e., how common versus rare the manifestations at this distance are). Below, mean ± std for each group reflect statistical differences by condition; marker area is proportional to *n* of that condition.

The Hamming-style distance metric applied to each individual’s manifestation revealed that the common manifestations had significantly lower distances from *M*_0_ than did the rare ones (mean distance of *M*_0_ to common: 2.2; mean distance of *M*_0_ to rare: 4.9; Kruskal–Wallis: *p* = 1.4*e* ^*−*2^; distance in Figure 2, relative relationship in Figure 4B).

PCA revealed that the majority of manifestations from the same branch as *M*_0_ were tightly clustered around *M*_0_. Alternate-branch manifestations appeared around the edge of the PCA projections (Figure 4). Moreover, alternate branches and Hamming-style distance values were highly correlated, with alternate branching becoming more likely as distance values increased. Additionally, as distance value increases, the proportion of common manifestations at a given distance value decreases (Distance vs. Commonality: *r* ^2^ = 7.6*e* ^*−*1^, *p* = 7.2*e* ^*−*9^; Distance vs. Alternative Branching: *r* ^2^ = 7.4*e* ^*−*1^, *p* = 3.0*e* ^*−*8^; Figure 4).

### 3.3 Manifestation rarity and reported symptom density

**FIGURE 5:**
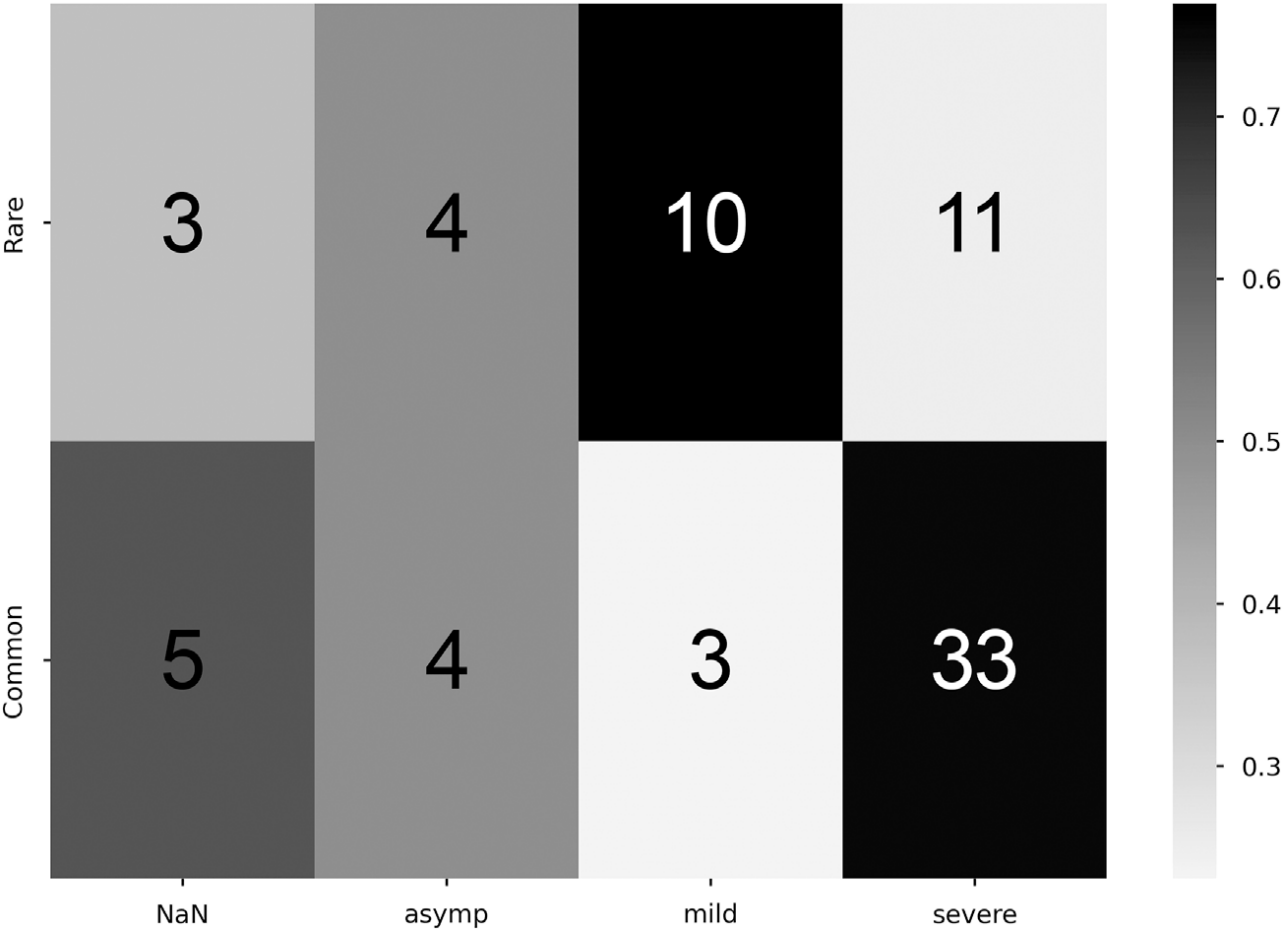
Correlation between commonality and symptomaticity of illness. Higher proportion values denote stronger correlation (colorbar, proportion Y commonality of X severity). Cell numeric label: total number of subjects within the given cell.

Student-T simulations were used to aid in estimating correlations between manifestation categorization (common/rare) and symptom density, performed with both the zeroth- and first-order maximum entropy distributions to set the prior odds as either uniform (50/50), or mean-matching values (∼60/40), respectively (see Table 1). For both versions of prior odds, we found significant departures from the prior odds for the rare manifestation and mild symptom density combination (Zeroth: *p* = 4.8*e* ^*−*2^; First: *p* = 5.5*e* ^*−*3^), and for the common and severe pairing (Zeroth: *p* = 5.0*e* ^*−*4^; First: *p* = 4.6*e* ^*−*2^) (Figure 5).

**TABLE 1:** *P* values associated with Student-T simulations for analysis of statistical significance of commonality ratios associated with symptom class densities.

Symptom density class	*p* (Zeroth)	*p* (First)
NaN	3.6*e* ^*−*1^	6.3*e* ^*−*1^
Asymptomatic	6.4*e* ^*−*1^	3.7*e* ^*−*1^
Mild	**4**.**8e** ^*−* **2**^	**5**.**5e** ^*−* **3**^
Severe	**5**.**0e** ^*−* **4**^	**4**.**6e** ^*−* **2**^

Bold face indicates statistically significant at the significance level of *α* = 0.05.

### 3.4 Manifestation rarity and participant sex

**FIGURE 6:**
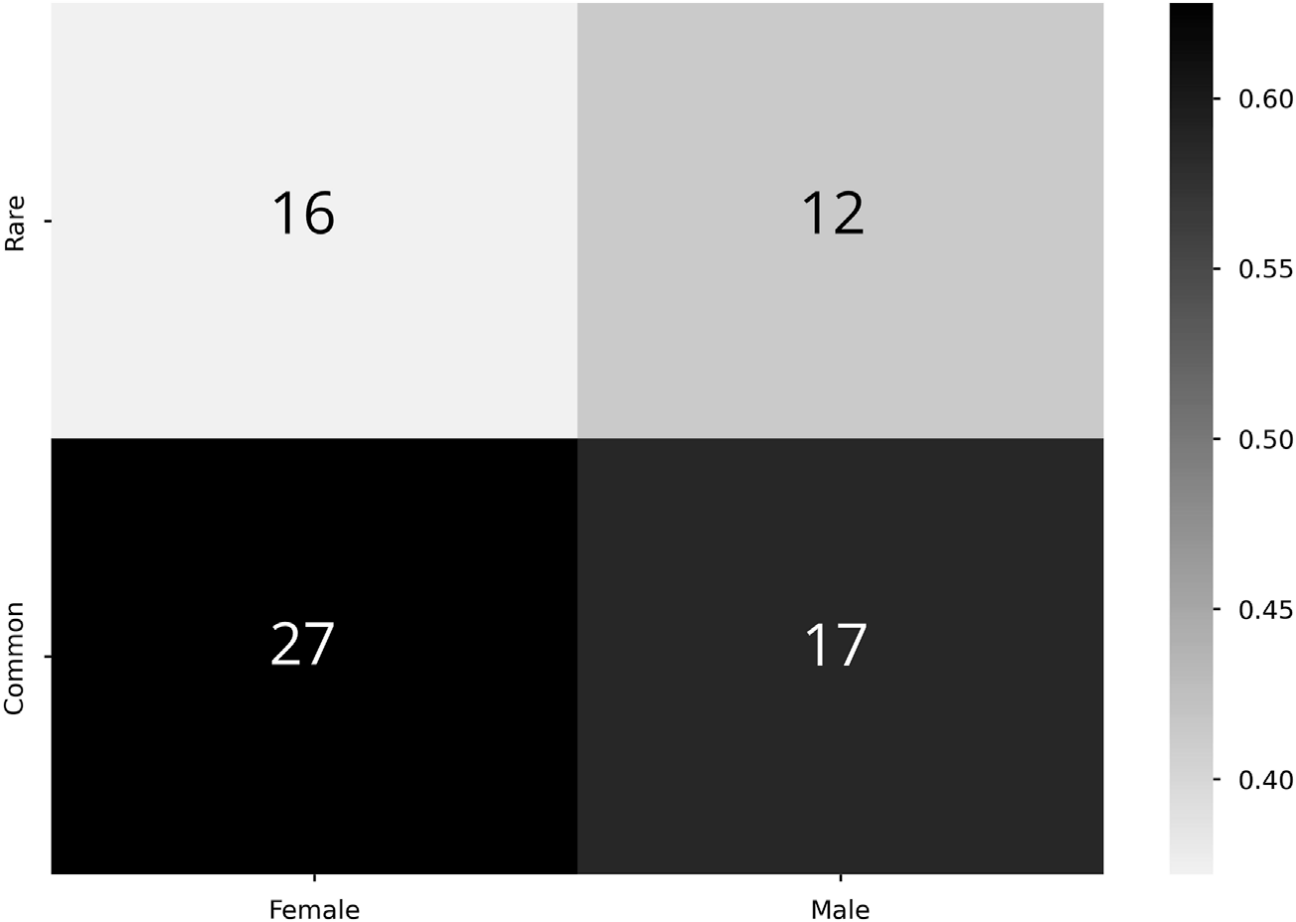
Correlation between commonality and sex. Higher proportion values denote stronger correlation (colorbar, proportion Y commonality of X severity). Cell numeric label: total number of subjects within the given cell.

Using the same process as the previous section, Student-T simulations were used to estimate correlations between manifestation categorization and sex of the participant, again performed with both the zeroth- and first-order maximum entropy distributions (see Table 2). For the zeroth-order maximum entropy distribution simulation, we found significant departures from the prior odds for females and the common manifestation (*p* = 3.2e^−2^); however, upon utilizing the first-order prior odds, this relationship become not statistically significant (*p* = 8.7e^−1^). For both versions of prior odds, there was no statistically significant relationship between males and either common or rare manifestations (Zeroth: *p* = 1.3*e* ^*−*1^; First: *p* = 5.6*e* ^*−*1^) (Figure 6).

**TABLE 2:** *P* values associated with Student-T simulations for analysis of statistical significance of commonality ratios associated with sex.

Sex	*p* (Zeroth)	*p* (First)
Female	**3.2*e*** ^***−*2**^	3.8*e* ^*−*1^
Male	1.3*e* ^*−*1^	5.6*e* ^*−*1^

Bold face indicates statistically significant at the significance level of *α* = 0.05.

## 4 Discussion

Our findings support the use of information-theoretic statistical approaches to quantify differences between physiological manifestations of illness. The approach allows for categorizing manifestations based on likelihood of appearance and developing relational trees across different observed manifestations. The notion of differing physiological “manifestations” has been explored before under broader contexts through concepts such as endotype (Kuruvilla et al., 2019) or physiotype (Ren et al., 2022). We found that an approach motivated by information-theoretic considerations building on this concept led to meaningful results that others may find useful.

The space of the 41 unique manifestations observed was smaller than the total space of possibilities (3^5^ = 243) and the theoretical upper bound of possibilities (*N* = 73; i.e., 1 per illness). This result cannot simply be explained by under-sampling and supports the hypothesis that the true structure of the system requires fewer dimensions than would a random permutation of all possible label results. Moreover, we found that not all manifestations are equally likely. Instead, the few most common manifestations cover a majority of cases, and these tend to be similar, clustering around a single physiological template, or cladistic-style branch. Figure 3 shows this idea that by viewing manifestations through the lens of this branch structure, not all illnesses that are different in one way need to be treated as wholly new. Instead, meaningfully different branches might be identified, and correlated to treatment choices.

Both the mild and severe symptom density classes had a statistically significant relationship to rarity. Rare manifestations were more likely to be associated with a mild symptom density class, whereas common manifestations were more likely to have a severe symptom density class, as seen in Table 1. These common manifestations mostly conform to the clinically expected physiological template of acute illness (e.g., elevated temperature, elevated heart rate, etc.) (Li et al., 2017). However, the appearance of anomalies among common manifestations that did not fit this expected template (evident in Figure 4B) highlights the potential importance of numerical techniques that can differentiate cases using physiologic data, as different physiological responses may have clinical relevance, indicating the need for different interventions. We were especially interested to see the apparent peak in physiological space (the largest dot in Figure 4A) with slopes down to a sparse, distributed plane of rare alternative manifestations. This suggests that there is likely one main peak, but that higher resolution analyses of larger data sets might reveal topological relationships that could highlight families of response type to different conditions. If this is proven true, then the techniques we develop here could help support precision in clinical decisions when such decisions can be supported by longitudinal data.

Additionally, these correlations reveal that asymptomatic cases show up with equal frequency in both common and rare manifestations, as seen in Figure 5. It is possible that this is an artifact of self-selection, whereby individuals with mild cases are less likely to seek testing. Taken at face value, it suggests that someone’s reporting of the absence of a symptom might be uncorrelated to the presence of a physiological sign for that symptom; this is consistent with early observations that many COVID-19 positive individuals had severely reduced blood oxygen upon admission to hospitals while reporting no shortness of breath (Couzin-Frankel, 2020). If this lack of correlation is verified in other datasets, it would both call for the adoption of continuous physiological measurement for illness detection and classification, and suggest the need for further study into the cause of asymptomatic individuals either not being aware of or reporting physiological changes. The presence of asymptomatic individuals who were unaware that their body was undergoing severe physiological changes highlights a potential psychological axis to COVID-19 illness.

To further showcase the use of this method, our analysis of the relationship between sex and manifestation categorization emphasizes alternative use cases to the method than symptom density. We find a statistically significant result using zeroth-order prior odds between females and common manifestations (Table 2). However, we caution readers against over interpretation of this result due to female-biased samples, with 43 out of the N = 73 (Figure 6). The loss of statistical significance when considering first-order prior odds adds to this point, as it shows that it is likely a statistically insignificant portion of the more frequently appearing sex (females) in the more frequently appearing manifestation category (common). Nevertheless, this exploration furthers that our method could be used to support similar investigations in future studies with larger population numbers.

Importantly, several caveats are notable in this context of this framework. First, we did not have a large enough sample size to conclude that this particular mapping of physiological data is representative of the entire COVID-19-positive population - that is, we make no claim that these are ideal or template manifestations of COVID-19, only that these manifestations appear substantially non-random in this data set, and that this technique appears adequate to reveal such patterns. Another caveat is that the COVID-19 strains being studied here are early strains of the virus, with all data collected in 2020. Thus, results pertaining to symptom density may not replicate in data from other COVID variants. Moreover, using the same process as described in this paper, we could have studied other factors such as age and sex. However, we chose to focus on symptom density on the assumption that symptom reports are to some degree independent from physiological measurements under examination. This particular investigation reveals that continuous physiological data contain a non-negligible amount of information regarding the symptom experience of a patient. Additionally, different devices will no doubt provide different modalities of physiological data in the future. Our approach here is device agnostic, and could be applied to any dataset of multimodal physiological data. As the number of modalities measured increases, the resolution of possible manifestations is expected to increase in proportion.

Beyond this, there could be an issue of self-selection, as participants in this study may not be representative of the entire population, and people who chose and were able to access COVID-19 testing in 2020 may differ from people who were infected but not able or willing to confirm their infection status through testing. Differences in manifestations across more diverse populations and the interactions of baseline “physiotypes,” or “physiolotypes” (Ivanov, 2021), with specific illness manifestations must be fleshed out before approaches using our framework could be of reliable utility. Additionally, different physiological metrics might show different relationships to different specific symptoms. As such, different sensors would likely yield somewhat different specific numbers in our results. These specific relationships are beyond the scope of our analyses here, however. Despite these important considerations, the methods, as a proof of concept, support the use of our information-theoretic framework for manifestation quantification. One of the “grand challenges” in network physiology is the detection of clusters within “physiolomes” (Ivanov, 2021), analogous to genotyping for risk. Although we use COVID-19 manifestations as a test for our hypothesis about non-random assortment of nodes in a network, from an information point of view, this approach may help contribute to this challenge. We recently showed in sleep that network models reveal additional information about illness risk beyond means or sums (Viswanath et al., 2024), and our methods described here further support the framework of mining for network states as a way to gain insight when dealing with physiological systems, especially when capturing dynamics over time within individuals.

In conclusion, we find that COVID-19 infection does seem to present in multiple specific, non-random physiological manifestations, and these manifestations are able to be mapped and classified from continuous physiological data. With sufficiently large samples, such approaches could be more widely validated. If successful at that point, these tools might enable faster identification of illness manifestation types in individual cases. This could not only aid in the selection of more specifically targeted COVID-19 treatment protocols tailored to particular physiologic manifestations, but it also seems likely that the same analytic approach may be useful in other illnesses as well.

## Data Availability

The data analyzed in this study is subject to the following licenses/restrictions: Oura’s data use policy does not permit us to make wearable device data (collected via the Oura Ring) data available to third parties. We can make self-report data available; please contact authors AM and BS to obtain an application to obtain these data.
